# Localized Outbreak of Macrolide-Resistant Pertussis in Infants, Japan, March–May 2025

**DOI:** 10.3201/eid3201.250824

**Published:** 2026-01

**Authors:** Takafumi Obara, Kyoko Kano, Takashi Yorifuji, Kohei Tsukahara, Naoki Yogo, Yuichiro Muto, Tetsuya Yumoto, Hiromichi Naito, Atsunori Nakao, Katsuki Hirai

**Affiliations:** Japanese Red Cross Kumamoto Hospital, Kumamoto, Japan (T. Obara, K. Kano, N. Yogo, Y. Muto, K. Hirai); Okayama University, Okayama, Japan (T. Obara, T. Yorifuji, K. Tsukahara, T. Yumoto, H. Naito, A. Nakao)

**Keywords:** pertussis, bacteria, antimicrobial resistance, outbreak, macrolides, drug resistance, infants, newborns, Japan

## Abstract

A localized pertussis outbreak involving 10 unvaccinated infants occurred in Kumamoto, Japan, during March–May 2025. Nine infants were admitted to the pediatric intensive care unit, 6 of whom received a confirmed diagnosis of macrolide-resistant *Bordetella pertussis* infection. This outbreak highlights the importance of booster vaccinations and resistance surveillance.

Since the relaxation of COVID-19–related public health measures, health officials have observed increased pertussis notifications in some regions of Japan. By April 2025, reported cases had already exceeded the previous year’s total. Alerts from the Japan Pediatric Society and the National Institute of Infectious Diseases have raised concerns about the rising incidence and spread of macrolide-resistant *Bordetella pertussis* (MRBP) (*1*,*2*), which has increased in East Asia, particularly in China, since around 2008 and more recently in Japan in 2024 (*3*,*4*). Nonetheless, clinical reports describing localized increases in pertussis incidence and the impact of MRBP on critical illness in infants remain scarce.

During late March through early May 2025, we identified an outbreak in Japan involving 10 unvaccinated infants < 2 months of age with PCR-confirmed pertussis. All infants were born at term and without perinatal complications. Nine required admission to the pediatric intensive care unit (PICU) for respiratory failure; 6 of those infants had confirmed MRBP infection. We conducted a retrospective descriptive analysis to characterize clinical features and examine potential association with regional pertussis trends.

Kumamoto Prefecture, in central Kyushu in western Japan, spans ≈7,400 km^2^ and had a population of around 1,690,000 in fiscal year 2024, including 210,000 children (<15 years of age). The region has single PICU, an 8-bed general unit at the Japanese Red Cross Kumamoto Hospital, which provides emergency critical care for children. Since January 1, 2018, pertussis has been classified in Japan as a category V infectious disease, requiring physician reporting of confirmed cases. We diagnosed pertussis at admission using the BIOFIRE FILMARRAY Respiratory Panel (bioMérieux, https://www.biomerieux.com). We performed antimicrobial susceptibility test or 23S rRNA gene sequencing to estimate macrolide susceptibility of infected strains. We extracted data from medical records and conducted analysis with Stata 19 (StataCorp LLC, https://www.stata.com).

We investigated annual cumulative pertussis cases in Kumamoto Prefecture (all ages, since 2018) together with infant PICU admissions at Japanese Red Cross Kumamoto Hospital (Figure) (*5*). During the 2025 outbreak, both the number and proportion of PICU admissions were higher than in previous years. All 9 infants had identifiable sick contacts within their household, most frequently school-aged siblings (Table). Six patients received azithromycin (5-day oral treatment) before PICU admission. After the fourth PICU admission, we tested nasopharyngeal swab specimens from the subsequent 6 infants by using bacterial culture or genetic analysis. One sample tested positive for *B. pertussis* on culture, showing erythromycin resistance. The remaining 5 samples underwent direct DNA sequencing of the *B. pertussis* 23S rRNA gene, revealing the A2047G mutation in all cases. The remaining infant, not in PICU, had a macrolide-sensitive strain. 

**Figure:**
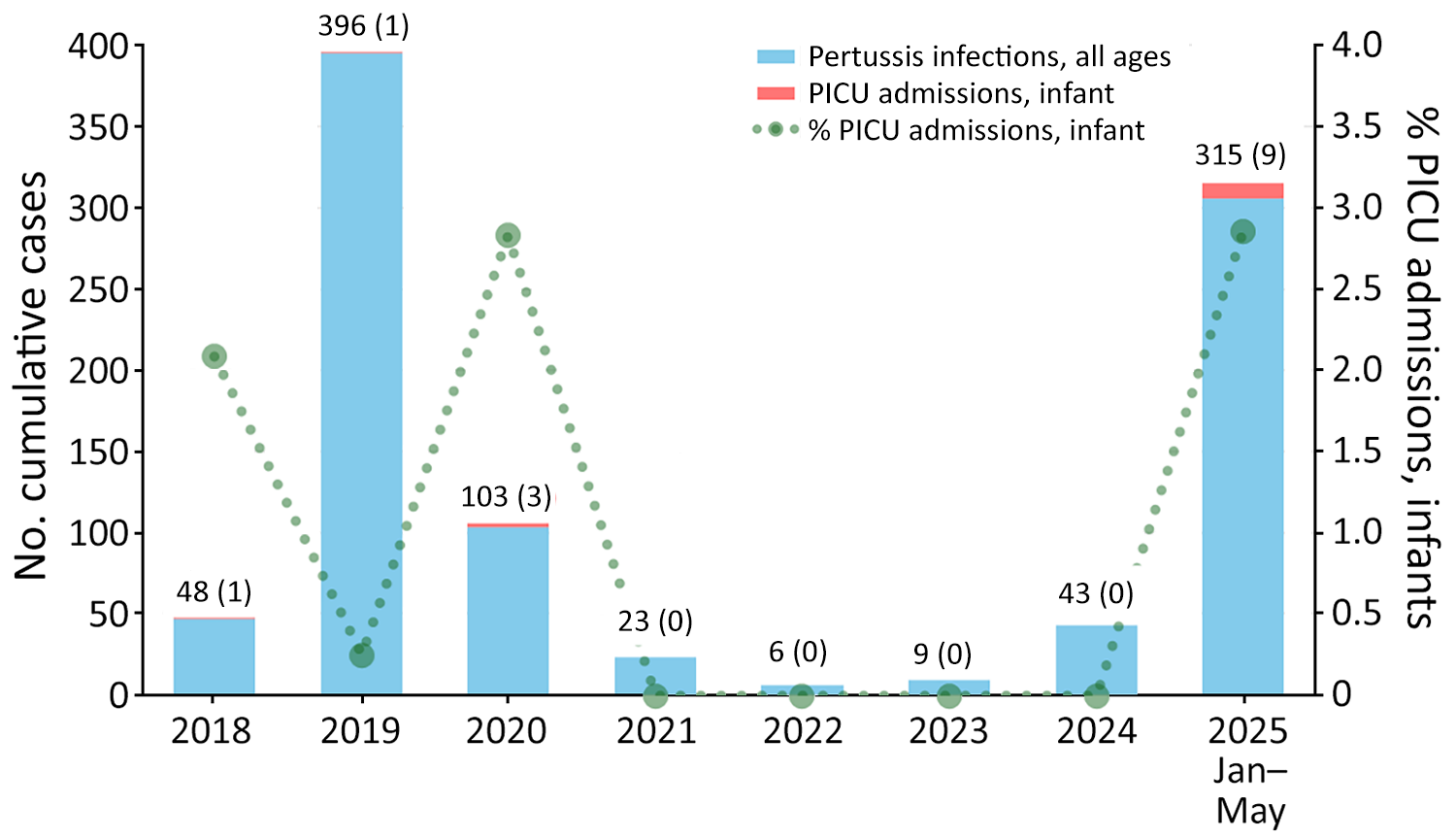
Cumulative reported pertussis cases (all ages) in Kumamoto Prefecture and infant PICU admissions at Kumamoto Red Cross Hospital, from study of a localized outbreak of macrolide-resistant pertussis in infants, Japan, March–May 2025. Reported pertussis cases are based on mandatory notifications (all ages) in Kumamoto Prefecture. PICU data represent infants admitted to the single regional PICU. Numbers above bars indicate total cases (PICU admissions). Data for 2025 are through May, and therefore interpretation of the proportion of PICU admissions relative to total cases should be made with caution. PICU, pediatric intensive care unit.

**Table:** Demographic information for 9 infants diagnosed with pertussis and admitted to the pediatric intensive care unit, Kumamoto, Japan, March–May 2025*

Variable	Value
Patient characteristics	
Median age, d (IQR)	44 (33–50)
Sex	
M	3 (33.3)
F	6 (66.7)
Median weight, g (IQR)	4,000 (3,600–4,266)
Median birthweight, g (IQR)	3,032 (2,560–3,190)
Median gestational age at birth, wk (IQR)	38 (37–39)
Underlying condition	0 (0.0)
Unvaccinated	9 (100)
Sick contact in family	9 (100)
Age group of symptomatic family members	
Siblings age 1–6 y	1 (11.1)
Siblings age 7–12 y	5 (55.6)
Parents age ≥18 y	3 (33.3)
Azithromycin use before PICU admission	6 (66.7)
MRBP confirmed†	6 (66.7)
Ventilator management	9 (100)
Nitric oxide therapy	1 (11.1)
Prone positioning	4 (44.4)
Rocuronium	8 (88.9)
Vasopressor	3 (33.3)
TMP/SMX use	6 (66.7)
Leukapheresis	3 (33.3)
Pulmonary hypertension diagnosis	2 (22.2)
Clinical course and outcomes	
Median days from symptom onset to PICU admission (IQR)	7 (7–12)
Median days from PICU admission to intubation days, d (IQR)	1 (0–1)
Median days of ventilator use, d (IQR)	8 (7–9)
Median PICU admission, d (IQR)	15 (14–19)
Median hospitalization, d (IQR)	23 (19–24)

*Values are no. (%) except as indicated. IQR, interquartile range; MRBP, macrolide-resistant *Bordetella pertussis*; PICU, pediatric intensive care unit; TMP/SMX, trimethoprim/sulfamethoxazole. †Of 6 infants tested (MRBP undetermined in 3 infants).

All PICU patients required intubation a median interval of 1 day after admission. The most frequent indications were paroxysmal coughing with desaturation and bradycardia. Most infants had persistent coughing and elevated airway resistance after intubation; 8 of 9 required continuous neuromuscular blockade. Six infants received oral trimethoprim/sulfamethoxazole for ≤14 days without adverse events, all from the fourth PICU case onward after MRBP identification. We did not give intravenous antibiotics prophylactically but initiated them after respiratory management for suspected secondary bacterial infection, including ventilator-associated pneumonia; 7 of 9 patients received ampicillin or ampicillin/sulbactam. Leukocytosis (>50,000 cells/μL) occurred in 3 infants, all treated with leukoreduction. Two patients developed pulmonary hypertension; 1 received inhaled nitric oxide. Median ventilation duration was 8 days and PICU stay 15 days for the PICU patients, longer than in our prior pertussis PICU cases (13 cases during 2012–2024). All patients survived without neurologic sequelae.

The temporal clustering of severe pertussis cases over a 2-month period suggests that increased community transmission contributed to the rise in critical cases. Although surveillance in Japan has reported sporadic MRBP cases, no prior studies have described a localized clinical outbreak. Despite the lack of prefectural-level susceptibility data, all 6 tested PICU cases were MRBP-positive, suggesting that a substantial proportion of circulating strains during this outbreak were resistant.

Pertussis most frequently affects children but also occurs in adults and adolescents. Outbreaks have been reported in fully vaccinated populations because of waning immunity, typically 5–10 years after vaccination (*6*,*7*). However, Japan does not currently require pertussis boosters beyond early childhood. From a public health perspective, a layered approach is essential: timely infant vaccination per the national schedule, booster doses for school-aged children to address waning immunity, and maternal tetanus-diphtheria-pertussis immunization during pregnancy to provide passive neonatal protection. Together, those strategies may help reduce secondary transmission within households and mitigate severe outcomes.

In severe infant pertussis, elevated leukocyte counts and pulmonary hypertension are associated with poor outcomes, underscoring the need for early monitoring and critical initiating of care-equipped setting (*8*–*10*). In the outbreak we describe, prolonged mechanical ventilation and extended PICU stays intermittently filled all PICU beds with MRBP-infected infant, placing a disproportionate burden on pediatric critical care capacity. This experience highlights how even localized outbreaks can strain systems in regions with only 1 PICU. In pertussis-endemic areas, considering antimicrobial-resistant strains, including MRBP, may support timely treatment decisions and improved outcomes.
